# Notes on the Ecology and Distribution of Species of the Genera of *Bondarzewiaceae* (*Russulales* and *Basidiomycota*) with an Emphasis on *Amylosporus*

**DOI:** 10.3390/jof10090625

**Published:** 2024-09-01

**Authors:** Shah Hussain, Moza Al-Kharousi, Dua’a Al-Maqbali, Arwa A. Al-Owaisi, Rethinasamy Velazhahan, Abdullah M. Al-Sadi, Mohamed N. Al-Yahya’ei

**Affiliations:** 1 Department of Plant Sciences, College of Agricultural and Marine Sciences, Sultan Qaboos University, P.O. Box 34, AlKhoud 123, Oman; shahpk85@gmail.com (S.H.); velazhahan@squ.edu.om (R.V.); 2 Oman Animal and Plant Genetic Resources Center (Mawarid), Ministry of Higher Education, Research and Innovation, P.O. Box 515, AlKhoud 123, Oman; 3 College of Agriculture, University of Al Dhaid, Sharjah P.O. Box 27272, United Arab Emirates

**Keywords:** *Amylosporus* section *Amylosporus*, *Amylosporus* section *Resupinati*, Oman, taxonomy

## Abstract

The family *Bondarzewiaceae* is an important and diverse group of macrofungi associated with wood as white rotting fungi, and some species are forest tree pathogens. Currently, there are nine genera and approximately 89 species in the family, distributed in tropical, subtropical, and temperate climates. To address the phylogenetic relationships among the genera, a combined ITS-28S dataset was subjected to maximum likelihood (ML), Bayesian inference (BI), and time divergence analyses using the BEAST package. Both ML and BI analyses revealed two major clades, where one major clade consisted of *Amylosporus*, *Stecchericium*, and *Wrightoporia austrosinensisa*. The second major clade is composed of *Bondarzewia*, *Heterobasidion*, *Gloiodon*, *Laurilia*, *Lauriliella*, and *Wrightoporia*, indicating that these genera are phylogenetically similar. *Wrightoporia austrosinensisa* recovered outside of *Wrightoporia*, indicating that this species is phylogenetically different from the rest of the species of the genus. Similarly, time divergence analyses suggest that *Bondarzewiaceae* diversified around 114 million years ago (mya), possibly during the Early Cretaceous Epoch. The genus *Amylosporus* is well resolved within the family, with an estimated stem age of divergent around 62 mya, possibly during the Eocene Epoch. Further, the species of the genus are recovered in two sister clades. One sister clade consists of species with pileate basidiomata and generative hyphae with clamp connections, corresponding to the proposed section *Amylosporus* sect. *Amylosporus*. The other consists of species having resupinate basidiomata and generative hyphae without clamps, which is treated here as *Amylosporus* sect. *Resupinati*. We provided the key taxonomic characters, known distribution, number of species, and stem age of diversification of each section. Furthermore, we also described a new species, *Amylosporus wadinaheezicus*, from Oman, based on morphological characters of basidiomata and multigene sequence data of ITS, 28S, and *Tef1-α*. With pileate basidiomata and phylogenetic placement, the new species is classified under the proposed *A*. sect. *Amylosporus*. An identification key to the known species of *Amylosporus* is presented. Ecology and distribution of species of the genera in the family are discussed.

## 1. Introduction

The family *Bondarzewiaceae* Kotl. and Pouzar was originally introduced to accommodate the wood-rotting mushrooms, with type genus *Bondarzewia* Singer [1]. The family is characterized by fleshy fruiting bodies with annual growth habit with porioid, hydnoid, or clavarioid hymenophore [1]. Later on, other genera, such as *Amylaria* Corner, *Amylosporus* Ryvarden, *Heterobasidion* Bref., and *Echinodontium* Ellis and Everh., were added to the family [2]. However, in several later studies, it was suggested that *Echinodontium* is sister to *Amylostereum* Boidin and has been treated under *Echinodontiaceae* Donk [3,4,5,6].

Macroscopically, *Bondarzewiaceae* is characterized by an annual to perennial growth habit with resupinate, effused-reflexed, pileate-sessile, pileate-stipitate to clavarioid fruiting bodies, and with smooth, tuberculate, poroid, and hydnoid hymenophore. Microscopically, species in the family have monomitic, pseudodimitic to dimitic hyphal systems, rarely trimitic hyphal systems, generative hyphae inamyloid, with or without clamps, skeletal hyphae inamyloid or dextrinoid (*Amylosporus*), gloeoplerous hyphae and gloeocystidia present or absent, and basidiospores usually asperulate to spinulose, verrucose to echinulate, hyaline to pigmented, and amyloid [7]. Currently, the family is composed of nine genera and 89 species [7,8,9]. Members of the family are widespread in distribution and found in tropical, subtropical, and temperate climates [7]. Ecologically, these species are mostly associated with wood as decaying fungi; however, some members, such as *Bondarzewia berkeleyi* (Fr.) Bondartsev and Singer, *B. montana* (Quél.) Singer, *Heterobasidion annosum* (Fr.) Bref., and *H. parviporum* Niemelä and Korhonen, are tree pathogens [6,10].

The genus *Amylosporus* was introduced in 1973, initially typified with *Amylosporus graminicola* (Murrill) Ryvarden [11]. Later on, *Amylosporus graminicola* was synonymized with *A. campbellii* (Berk.) Ryvarden, the latter which became the type species of the genus [12]. Initially, the genus was placed in Wrightoporiaceae Jülich [13], but recently it was treated under *Bondarzewiaceae* [7,8,9]. Morphologically, the genus is distinguished by annual to perennial basidiomata, which may pileate, effused-reflexed, or resupinate; the pileate form may be stipitate or sessile; cap surface buff to ochraceous or pinkish, darker at the center; pore surface whitish to pale pinkish or lilac to vinaceous; and pores 2–10 per mm, rounded to angular. Microscopically, the genus has a dimitic hyphal system; generative hyphae that are thin-walled, rarely branched, with simple septa; and clamp connections may be present or absent; skeletal hyphae are usually thick-walled, frequently branched, dextrinoid, or IKI−; gloeoplerous hyphae may be present or absent; clamp connections are absent in hymenium; cystidia are absent; basidia are usually four-spored rarely eight-spored; and basidiospores are mostly thin-walled, finely asperulate, IKI+, CB+, or CB− [13,14,15].

Currently, there are 13 species of *Amylosporus*, distributed in the Americas, Europe, Africa, and Asia; however, no member of the genus has been reported from Australia [15,16,17,18]. Although, several records of the genus from Australia are available on the GBIF website (https://www.gbif.org/species/, accessed 17 July 2024). Sequence data (ITS and 28S) are available only for eight species, which could be the reason that the species of this genus are not yet classified into sections. However, basidiomata form (resupinate/pileate) and the presence or absence of clamps in generative hyphae could be employed for the infrageneric classification of *Amylosporus*.

Species of *Amylosporus* are parasitic or saprotrophic in nature, associated with broad-leaved trees or found on the forest floor under grasses, probably associated with underground angiosperm wood [13,17]. The parasitic species are causing white rot in a number of angiosperm plants [19].

In this study, we presented a robust phylogeny and time divergence estimation of *Bondarzewiaceae*, representing all the genera of the family except the monotypic genus *Amylaria himalayensis* Corner, for which the sequence data are not available. For the first time, infrageneric classification of the genus *Amylosporus* with two proposed sections is provided. We also described a new species in *Amylosporus*, and a taxonomic key to known taxa of the genus is presented. Ecology and species distribution of the genera in the family are further discussed.

## 2. Materials and Methods

### 2.1. Study Sites, Sampling and Morphological Examination

**Figure 1:**
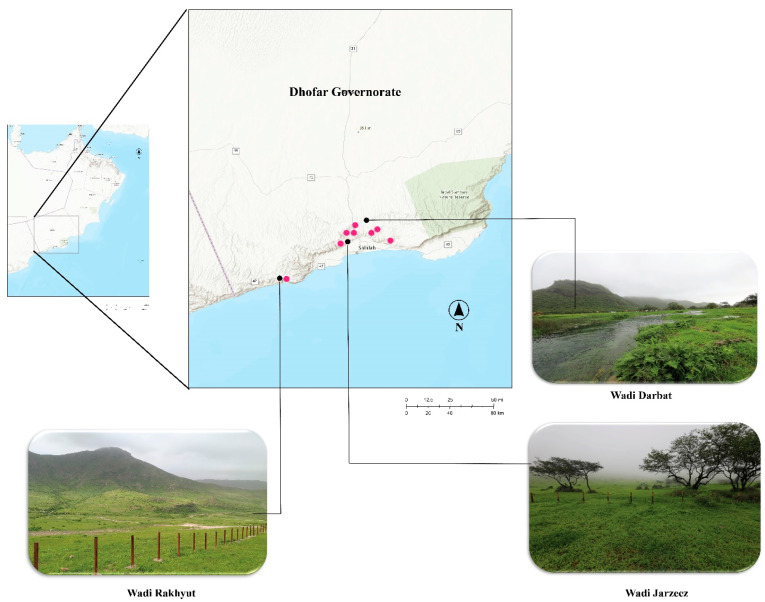
Map of the sampling sites, the Dhofar Governorate, located in south of Oman, the region consisting of lush wades and mountains, the photos showing some parts of the study area (Wadi Darbat with GPS coordinates: 17°07′ N, 54°43′ E; Wadi Jarzeez GPS coordinates: 17°13′ N, 54°05′ E; Wadi Rakhyut GPS coordinates: 16°80′ N, 53°42′ E). The pink dots represent sampling sites.

The study area, the Dhofar region (Figure 1), located in the south of Oman, experiences monsoon rains from mid-June to mid-September each year [20]. The sea-facing sides of mountains and plains of Dhofar changes into a lush-green region in the summer, with the highest plant biodiversity in the entire Arabian Peninsula [21]. During the summer season, a number of mushroom-forming fungi appeared on the forest floor, on logs, tree trunks, on termite mounds, etc. Mushroom exploration expeditions are recently initiated in the region in which several new species have been described [22,23,24,25,26], including a polypore *Fuscoporia dhofarensis* Al-Sadi and S. Hussain [27].

Basidiomata were collected in the months of August-September during the years 2022 and 2023, from Dhofar Governorate, Oman. The specimens were found on the forest floor, under grasses. This mushroom is very common in the region during the monsoon season. Morphological characteristics, such as basidiomata size, shape, color, texture, etc., were noted in the field based on fresh collections. Basidiomata were photographed in the natural habitat, tagged, dried in a fruit dehydrator (45 °C temperature), and kept in zipper bags. The dried basidiomata were subjected to a low-temperature treatment at −80 °C for two weeks to kill all the insect’s eggs/larvae. Small, hand-made sections were prepared from the upper surface of the pileus, subiculum, tubes, and stipe for microscopic investigations. The sections were mounted in 5% aqueous KOH solution (*w/v*), rehydrated in 1% aqueous Congo red (*w*/*v*). Microscopic structures, such as basidiospores, basidia, generative hyphae, skeletal hyphae, etc., were examined under a compound microscope (ECLIPSE Ni-U, Nikon Co., Ltd., Minato City, Japan). The studied materials are deposited at Mawarid Center, AlKhoud, Muscat, Oman.

### 2.2. Molecular Identification

DNA extraction was achieved using the X-AMP DNA extraction kit (Dubuque, IA, USA), according to the manufacturer’s protocol. Three DNA regions were amplified, which included the internal transcribed spacer region (ITS1-5.8S-ITS2 = ITS), the D1/D2 domain of the large subunit of nuc rDNA (28S), and a portion of the translation elongation factor 1 alpha (*Tef1-α*) gene. The following primers were used: ITS1F/ITS4 for ITS [28,29], LR0R/LR5 for 28S [30], and EF1-983/EF1-1567R for *Tef1-α* [31], respectively. The PCR reactions and conditions were according to Chen et al. [32]. Purification and sequencing of the PCR products was performed at Macrogen Inc. © (Seoul, Republic of Korea).

### 2.3. Phylogenetic Analyses

Only ITS and 28S sequence data are available (in GenBank) for most species in *Bondarzewiaceae*; therefore, we employed the sequences of these two regions to address the phylogeny of the family. A combined ITS-28S dataset was constructed from 52 specimens including *Amylonotus labyrinthinus* (Yuan 1475) as the outgroup taxon. The dataset represents 51 specimens of *Bondarzewiaceae*, including 24 samples of *Amylosporus*, 12 of *Wrightoporia*, three specimens of each *Bondarzewia* and *Heterobasidion*, and two specimens of each *Gloiodon* P. Karst., *Laurilia* Pouzar, *Lauriliella* S.H. He and Nakasone, *Stecchericium* D.A. Reid. Only *Amylaria* was not included because no sequence of the genus is available in GenBank. The dataset was aligned using the online version of the multiple sequence alignment tools (MAFFT v. 7 [33]), applying the L-INS-I strategy, and then manually adjusted in BioEdit v. 7.0.9.0 [34]. Details of the specimens included in the phylogenetic analyses are given in Table 1.

Two methods of phylogenetic analyses were used: maximum likelihood (ML) and Bayesian inference (BI). The ML analyses were executed with RAxML-HPC BlackBox (https://www.phylo.org/portal2/; [35]), using the CIPRES Science Gateway platform (https://www.phylo.org/portal2/; accessed on 25 July 2024 [36]). The best model was (TPM2u+F+I+G4) selected using jModelTest2 [37]. Branch support for the ML phylogeny was calculated using 1000 bootstrap replicates. Similarly, for BI analyses, BEAST v. 1.8.2 [38] was used. Initially, the *FASTA* file was converted to a *nexus* datafile using ClustlX v. 2.1 [39]. The *nexus* datafile was converted to an *XML* file using BEAUti v. 1.8.2 [38]. The model of speciation chosen was the Birth-Death Incomplete Sampling model [40]. Four separate runs were performed with BEAST on the XSEDE tool (v. 1.8.2) on the CIPRES Science Gateway [36]. The *log* files were traced in Tracer v. 1.6 [41] to check the effective sample size (ESS) values for all parameters. The ESS value for each parameter was well over 200. The tree files were combined in LogCombiner v. 1.8.2 [42]. A maximum clade credibility (MCC) tree was obtained using the TreeAnnotator v. 1.8.2 [42]. Values were considered significant when the ML bootstrap (BT) percentage was ≥70 and BI posterior probabilities (PPs) was ≥0.95. FigTree 1.4.2 [43] was used for tree visualization, and the tree was annotated using Adobe Illustrator CC2019.

### 2.4. Estimation of the Stem Age of Amylosporus

To estimate the stem age of diversification of *Amylosporus* within *Bondarzewiaceae*, the combined ITS-28S dataset, comprising 52 specimens, was used. This dataset represents all the representative genera of the family except *Amylaria*, for which no molecular data are available. The time divergence analyses were estimated using BEAST v. 1.8.2 [38]. An *XML* file was constructed using BEAUti v. 1.8.2 [42], where substitution models were optimized. A *Yule* speciation model was chosen [44] with HKY+G+I distribution, following ModelFinder [45]. The *ulcd.mean* parameter was specified with a uniform distribution, where the initial value was 0.033, the higher set was 1.0, and the lower set was 0.0, using a lognormal relaxed molecular clock. For *treeModel.rootHeight* parameter, a normal distribution with a mean age of 117 was specified (standard deviation 10) following He et al. [7]. Four independent runs of MCMC chains were run, with 20 million generations each, a logging state of 1000 generation, and discarding the first 10% as burn-in. *Log* files were checked in Tracer v. 1.6 [41]. Using TreeAnnotator v. 1.8.2 [42], the *Tree* files were merged into a maximum clade credibility (MCC) tree.

The alignment files were submitted to TreeBase (https://www.treebase.org/treebase-web/home.html; http://purl.org/phylo/treebase/phylows/study/TB2:S31306; accessed on 8 June 2024).

## 3. Results

### 3.1. Molecular Phylogenetic Analyses

**Figure 2:**
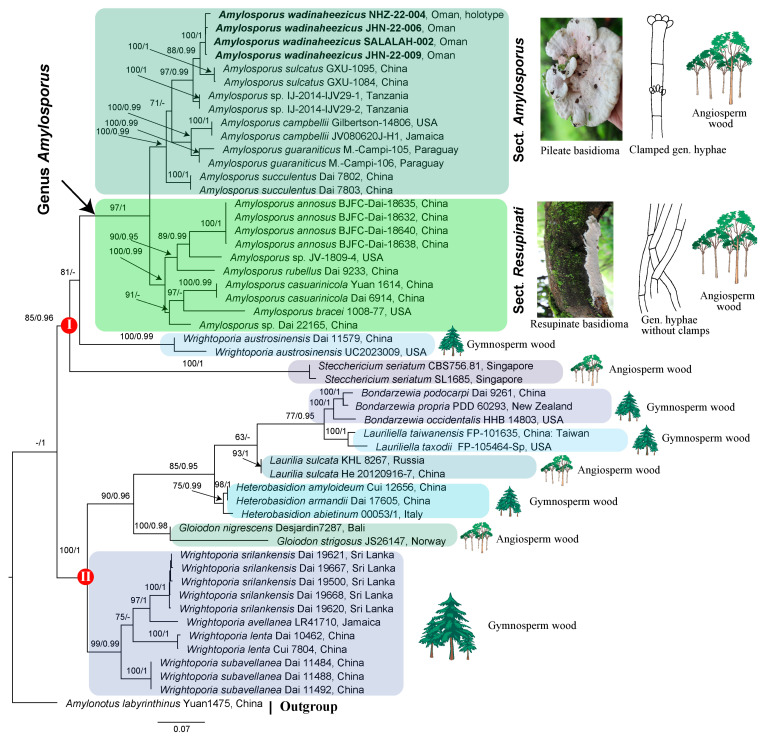
Maximum likelihood phylogeny of family *Bondarzewiaceae* based on combined ITS-28 sequences; values above the nodes are maximum likelihood bootstrap (BT) and Bayesian posterior probabilities (PPs), with *Amylonotus labyrinthinus* (Yuan 1475) as outgroup. Species within the family were recovered in two clades. Clade-I consists of genus *Amylosporus*, *Stecchericium* and a species of *Wrightoporia W. austrosinens*, with moderate ML (BT 85%) and excellent BI support (PPs 1). Species in *Amylosporus* are further split into two sister clades: one sister clade with species having pileate basidiomata and clamped generative hyphae, representing the proposed section *A*. sect. *Amylosporus*; and the other sister clade with taxa having resupinate to effused-reflexed basidiomata and generative hyphae without clamp connections, making the section *A*. sect. *Resupinati*. Clade-II consisted of genera: *Bondarzewia*, *Heterobasidion*, *Gloiodon*, *Laurilia*, *Lauriliella*, and *Wrightoporia*, with excellent phylogenetic support in both analyses (BT 100%, PPs 1). The associated wood type, either angiosperm or gymnosperm are represented with their respective tree icon. Sequences of the new species *Amylosporus wadinaheezicus* are shown in bold font.

The combined ITS-28S alignment was 2044 characters long, including 1000 constant sites, 795 parsimony informative sites, and 249 uninformative sites. Both ML and BI analyses resulted phylogenetic trees with similar topologies. The phylogeny inferred from ML analyses is presented with values from both BT and PPs (Figure 2). Species within Bondarzewiaceae were recovered in two major clades. Clade-I corresponds to the genus *Amylopsorus*, *Stecchericium*, and one species of *Wrightoporia*, *W. austrosinensis*, with moderate support in ML analysis (BT 85%) and excellent support in BI analysis (PPs 0.96). Species in *Amylosporus* are further split into two sister clades, each with strong phylogenetic support (BT).

### 3.2. Divergence Time Estimation

**Figure 3:**
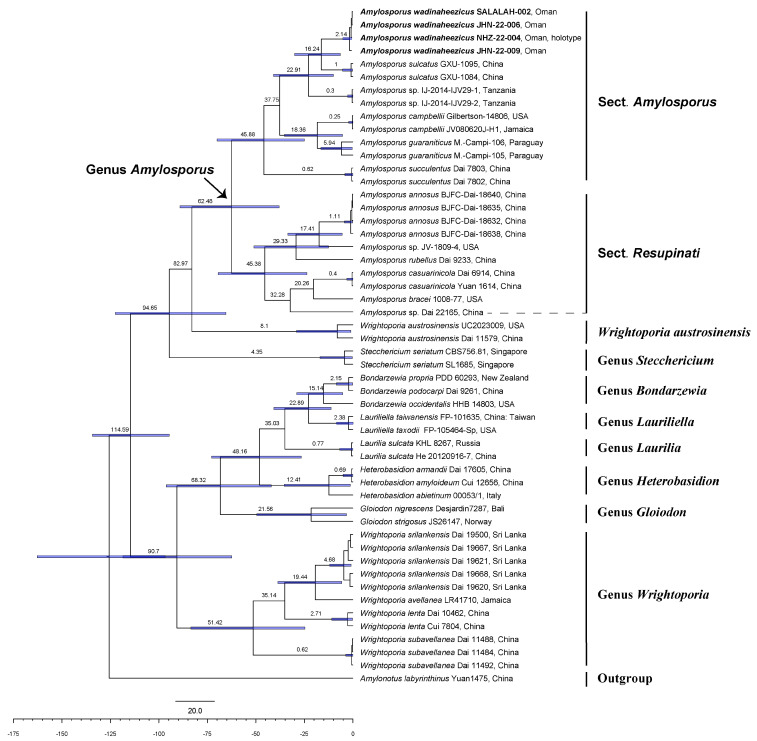
Maximum clade credibility (MCC) tree of Bondarzewiaceae obtained from BEAST analysis based on ITS-28S dataset comprises 52 specimens including the outgroup taxon *Amylonotus labyrinthinus* (Yuan 1475). The dataset represents all the genera which belonging to Bondarzewiaceae according to Index Fungorum and Outlines of Fungi, except *Amylaria*, for which no sequence data are available. Taxa in the family recovered into two clades, in congruence to ML and BI analyses. One clade with an estimated stem age of diversification of approximately 94 mya, consisting of genus *Amylosporus*, *Stecchericium*, and *Wrightoporia austrosinens*. The horizontal dashed line separate *Amylosporus* for the rest of the genera of the family. The other clade with estimated stem age of divergence around 90 mya, consisting of *Bondarzewia*, *Heterobasidion*, *Gloiodon*, *Laurilia*, *Lauriliella*, and *Wrightoporia*. The 95% highest posterior density of divergence time estimations is marked by horizontal bars.

Species of *Bondarzewiaceae* were diversified approximately 114 million years ago (mya); the results are presented in Figure 3. Similar to ML and BI phylogenies, genera in the family recovered in two major clades using the time divergence estimation analyses. One major clade consisting of *Amylopsorus*, *Stecchericium*, and *Wrightoporia austrosinensis*. The ancestors of these taxa are supposed to have diversified approximately 94 mya. The stem age of diversification of *Amylosporus* is estimated to be around 62 mya. The clade of *Amylosporus* is further split into two sister clades, each with an estimated diversification age around 45 mya. Similarly, the other major clade consisted of *Bondarzewia*, *Heterobasidion*, *Gloiodon*, *Laurilia*, *Lauriliella*, and *Wrightoporia.* The estimated stem age of the clade is approximately 90 mya.

### 3.3. Taxonomy

#### 3.3.1. *Amylosporus* Sect. *Amylosporus*

Type: *Amylosporus campbellii* (Berk.) Ryvarden, Norw. J Bot. 24: 217 (1977).

Etymology: The epithet of the section “*Amylosporus*” follows the name of the genus *Amylosporus*.

Key characteristics of the section: Basidiomata pileate and generative hyphae with clamp connections.

Morphological description: Basidiomata pileate and stipitate, growth habit mostly annual, rarely perennial, pore 2–6 per mm, pore surface whitish-cream to pinkish when fresh, slightly brownish to ochraceous when dry, generative hyphae with single and multiple clamp connections, gloeoplerous hyphae usually present, rarely absent. Ecologically, species of this section are associated with angiosperm wood, may cause wood rot, or are found saprotrophically under grasses.

Known distribution: Species in this section are wide-spread. *Amylosporus campbellii* is mainly a subtropical species and can be found in tropical and subtropical regions of Africa, southern North America, Central and South America, Southeast Asia [2,46,47,48,49], and temperate Europe [16]. *Amylosporus guaraniticus* is distributed in humid–subtropical region of Paraguay, South America, and found on buried logs [17]. *Amylosporus succulentus*, *A. sulcatus*, and the proposed new species *A. wadinaheezicus* are found in subtropical regions of Asia [14,18]. *Amylosporus deadaliformis* G.Y. Zheng and Z.S. Bi is a subtropical species, reported from Guangdong Province, southeast China [50]. Similarly, *Amylosporus auxiliadorae* Drechsler-Santos and Ryvarden, distributed in the tropical region of northeast Brazil [51]. Similarly, one unnamed species *A*. sp. (IJ-2014_IJV29-1, IJ-2014_IJV29-2), distributed in East Africa, and another unnamed *A*. sp. (BAB-5055) in India.

Notes: Currently there are seven named and two unnamed species in *A*. sect. *Amylosporus*, *viz*. *A. auxiliadorae, A. campbellii*, *A. deadaliformis*, *A. guaraniticus*, *A. succulentus*, *A. sulcatus*, *A. wadinaheezicus*, *A*. sp. (IJ-2014_IJV29-1, IJ-2014_IJV29-2), and *A*. sp. (BAB-5055). Sequence data (ITS and 28S) are available for all these species except *A. auxiliadorae* and *A. deadaliformis*.

Stem age and phylogenetic support: The estimated stem age of the section is approximately around 45 mya, and PPs is 1 in the MCC tree (Figure 3), strong phylogenetic support in both ML and BI analyses (BT 100%, PP 0.99; Figure 2).

#### 3.3.2. *Amylosporus* Sect. *Resupinati* S. Hussain, Al-Sadi, Al-Yahya’ei and R. Velazhahan, Sect. Nov.

MycoBank No: 853625

Type: *Amylosporus annosus* Y.C. Dai, P. Du, and X.H. Ji, Phytotaxa 424: 295 (2019).

Etymology: The epithet “*Resupinati*” refers to the resupinate type of basidiomata, which is a common character in the species of this section.

Key characteristics of the section: Basidiomata resupinate to effused-reflexed, and generative hyphae without clamp connections (except *Amylosporus bracei*).

Morphological description: Basidiomata resupinate or effused-reflexed, growth habit perennial or annual, pores 3–10 per mm, pore surface whitish-cream to yellowish, generative hyphae simple septate, multiple clamp connections only present in *A. bracei*, gloeoplerous hyphae usually present, rarely absent, basidia regularly four-spored, rarely eight-spored, basidiospores subglobse to ellipsoid. Ecologically, species of this section are associated with angiosperm trees, causing white rot; some species are wood decomposers.

Known distribution: Species in this section are distributed in tropical regions. *Amylosporus annosus* reported from tropical Southeast Asia, only known from its type of location, Malaysia [15]). *Amylosporus casuarinicola* is a subtropical species, reported from the southwest coastal region of Beihai, Guangxi, China [52]. *Amylosporus bracei* is a tropical species, distributed in different parts of the world, viz. Belize, Martinique Island, Central America; Sao Paulo, Brazil, South America; Florida, North America [13]. This is the only species in the section with clamped generative hyphae. *Amylosporus efibulatus* (I. Lindblad and Ryvarden) Y.C. Dai, Jia J. Chen, and B.K. Cui found in tropical Central America [13]. *Amylosporus rubellus* has been reported from Beijing, China, with a monsoon-influenced humid continental climate [13]. Similarly, *A. ryvardenii* Stalpers is another tropical species, reported from East Africa [53].

Notes: Currently there are six named species in the *A*. sect.: *Resupinati*, *viz*. *A. annosus*, *A. bracei*, *A. casuarinicola*, *A. rubellus*, *A. efibulatus*, and *A. ryvardenii*, and two unnamed species: *A*. sp. (JV-1809-4) from the USA [13] and *A*. sp. (Dai 22165) from China [52]. Sequence data (ITS and 28S) are available for the first four named species and the two unnamed species.

Stem age and phylogenetic support: The estimated stem age of the section is approximately 45 mya, with PPs of one in the MCC tree (Figure 3), and excellent phylogenetic support in both ML and BI analyses (BT 100%, PP 0.99; Figure 2).

#### 3.3.3. *Amylosporus wadinaheezicus* S. Hussain, Al-Sadi, Al-Yahya’ei, Al-Kharousi, and A. Al-Owaisi, sp. Nov. Figure 4 and Figure 5

MycoBank No: 853624

Holotype: Sultanate of Oman: Dhofar: Wadi Naheez, on soil probably on underground wood, below *Anogeissus dhofarica* trees, 7 August 2022, S. Hussain, A. Al-Owaisi, Al-Yahya’ei, and Al-Sadi, NHZ-22-004 (Holotype Mawarid-NHZ-22-004), GenBank accessions: ITS = PP681308, 28S = PP681313, *Tef1-α* = PP683472.

Etymology: The specific epithet ‘*wadinaheezicus’* refers to the holotype location Wadi Naheez, located in the south of the Sultanate of Oman.

Description: Basidiomata annual, pileate, centrally stipitate, laterally fused pielei, solitary, moist and juicy when fresh, in some specimens with a release of pinkish, juicy droplets, becoming corky and light in weight upon drying. The pileus is circular to semicircular, projecting up to 5 cm long, 15 cm wide, 3 cm thick at the base, with a depressed center, thinner towards the margins, margin undulating, obtuse. The pileus surface is creamy-whitish to pinkish, at the center dark pinkish to pale brownish, becoming pale yellowish on drying, cottony. The pore surface creamy to white when fresh, pale yellowish on drying, pores circular to angular, 2–4 per mm; sterile margin thin, indistinct, and up to 1 mm wide; dissepiment up to 150 µm thick, with lacerate mouth. The context is creamy to light pinkish, fleshy and moist in fresh conditions, pale brownish and corky when dry, and up to 2 cm thick. The tubes are whitish when dry, becoming pale brownish upon drying, and up to 1 cm long. Stipe 1–5 × 2–2.5 cm, stout, thick, pinkish to pale brownish when fresh, becoming brownish on drying, moist.

Smell pleasant when fresh, pungent when dried.

Hyphal system dimitic. Tramal generative hyphae simple septate, rarely branched, with single clamp connection, skeletal hyphae thick-walled, dextrinoid, CB+; contextual generative hyphae simple septate with multiple clam connections, skeletal hyphae frequently branched, think-walled, inflated in KOH. ***Context*** generative hyphae dominant, hyaline, with multiple clamp connections, thin-walled, frequently branched, 8.5–15.5 µm in diam; skeletal hyphae frequent, thick-walled with a narrow to wide lumen, branched, flexuous, hyaline, 3–9 µm in diam; gloeoplerous hyphae frequent, rarely branched, hyaline, thin-walled, simple septate, with multiple clamp connections, with granular or oily internal contents, 6–11 µm in diam. ***Tubes*** generative hyphae dominant, hyaline, thin-walled, rarely branched, with single clamp connection, 3–5 µm in diam; skeletal hyphae, hyaline, rarely branched, thick-walled, with wide lumen, 3–4.5 µm in diam; gloeoplerous hyphae absent; cystidia and cystidioles absent; basidia 40–58 × 7–9 µm, clavate, with a basal simple septum and four sterigmata, hyaline, smooth. ***Basidiospores*** oblong to ellipsoid, hyaline, thin-walled, finely asperulate, CB+, IKI+; (4.0) 4.5–5.0 (6.0) × (2.5) 3.0–3.5 (4.0) µm, Q = 1.4–1.7, av. Q = 1.5, average length × width = 5 × 3.3 µm (n = 90/4).

Habit, habitat, and distribution: Saprotrophic, solitary, under the trees (probably with the roots) of *Anogeissus dhofarica* A.J. Scott (family Combretaceae). Fruiting bodies of the fungus occurring during monsoon season, from July to September, widespread in Southern Oman.

**Figure 4:**
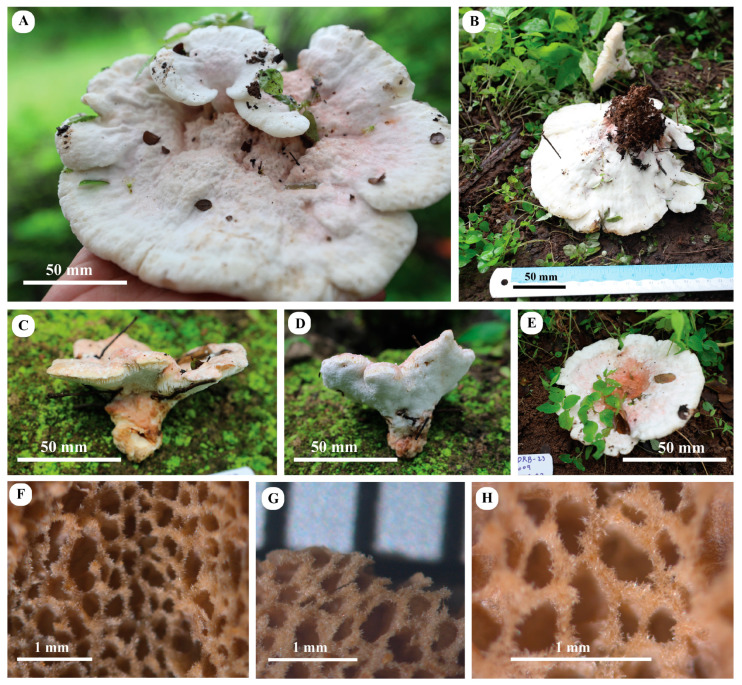
Basidiomata of *Amylosporus wadinaheezicus*, (**A**,**B**); Mature basidiomata NHZ-22-004 (holotype), (**C**,**D**); HOD-23-012, (**E**); and DRB-23-009, (**F**–**H**). Pore surface under stereo microscope (NHZ-22-004).

Additional specimens examined: SULTANATE of OMAN: Dhofar, Salalah, Wadi Naheez, below *Anogeissus dhofarica* trees, 7 August 2022, S. Hussain, A. Al-Owaisi, Al-Yahya’ei and Al-Sadi, NHZ-22-004a (Mawarid-NHZ-22-004a), GenBank accession: ITS = PP681309; Wadi Jahaneen, below *Anogeissus dhofarica* trees, 8 August 2022, S. Hussain, A. Al-Owaisi, Al-Yahya’ei and Al-Sadi, JHN-22-009 (Mawarid-JHN-22-009), GenBank accessions: ITS = PP681311, 28S = PP681314, *Tef1-α* = PP683473; same area and same date, S. Hussain, A. Al-Owaisi, Al-Yahya’ei and Al-Sadi, JHN-22-006 (Mawarid-JHN-22-006), GenBank accessions: ITS = PP681310, 28S = PP681316, *Tef1-α* = PP683475; Attin, below *Anogeissus dhofarica* trees, 6 September 2022, A. Al-Owaisi, SALALAH-002 (Mawarid-SALALAH-002); GenBank accessions: ITS = PP697985; 28S = PP681315, *Tef1-α* = PP683474; Wadi Darbat, below *Anogeissus dhofarica* trees, 26 August 2023, S. Hussain and M. Al-Jahwari, DRB-23-009 (Mawarid-DRB-23-009); Mirbat, on soil, under grasses, 21 August 2023, S. Hussain and M. Al-Jahwari, MRT-23-001 (Mawarid-MRT-23-001; Anghetat (the place of Prophet Hood’s tomb Peace Be Upon Him), on soil, with mosses, 27 August 2023, S. Hussain and M. Al-Jahwari, HOD-23-012 (Mawarid-HOD-23-012), GenBank accession: ITS = PP681312.

**Figure 5:**
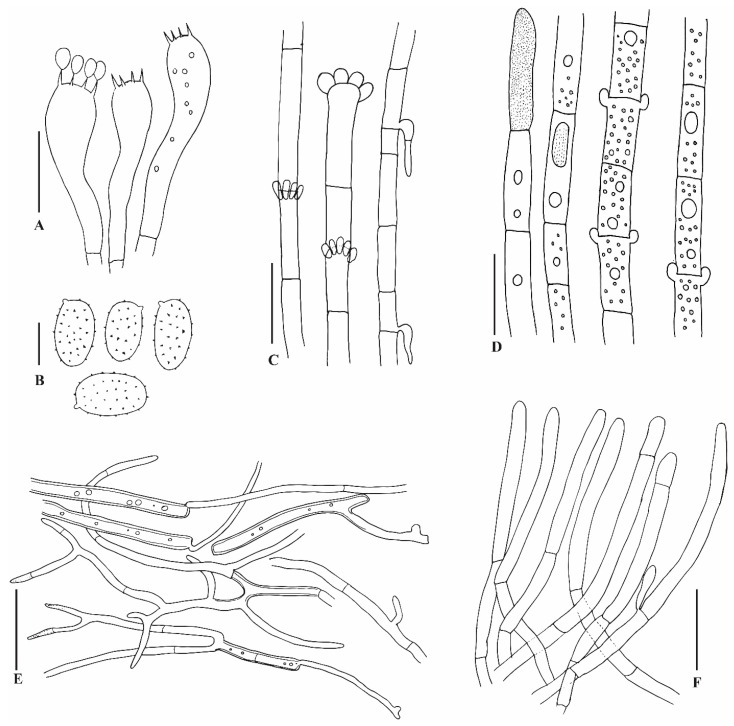
Illustration of anatomical characters of *Amylosporus wadinaheezicus* (holotype NHZ-22-004). (**A**) Basidia, (**B**) Basidiospores, (**C**) Contextual generative hyphae, (**D**) Gloeoplerous hyphae, (**E**) Contextaul skeletal hyphae, and (**F**) Tube generative hyphae. Scale bars: (**A**) = 10 µm, (**B**) = 5 µm, and (**C**–**F**) = 7 µm.

Notes: The new species is distinguished by a pileate stipitate basidiomata, fleshy and soft, pinkish cap surface with pale brownish to dark pinkish center, creamy to pinkish pore surface, simple septate and multi-clamped generative hyphae. The new species *Amylosporus wadinaheezicus* is widespread in Dhofar region, southern Oman, where it was found as saprotrophic on forest floor under grasses, open fields, pastures, simply everywhere in the area which is impacted by the monsoon season. Most probably, the species is associated with underground wood, or it only grows on roots of *Anogeissus dhofarica*, perhaps it could be an endemic species to this region. Using ML and BI analyses, *Amylosporus wadinaheezicus* fall in the clade consisting of the proposed *A*. sect. *Amylosporus*. Species in *A*. sect. *Amylosporus* share plileate basidiomata with creamy pinkish to pale brownish pileus surface, whitish to pale brownish pore surface, and clamped generative hyphae. *Amylosporus auxiliadorae* associated with angiosperm wood, with pale brownish pileus surface, pore surface pale yellowish, contextual skeletal hyphae shorter in diam (up to 7 µm), absence of gloeoplerous hyphae in context, basidiospores ellipsoid to ovoid, finely asperulate, measuring 4.0–5.0 × 2.5–4.0 µm [51]. This species is only known from type locality, northeast Brazil, and sequence data are not available for this species [51]. *Amylosporus succulentus* shares the pileate basidiomata and whitish pore surface with *A. wadinaheezicus*. *Amylosporus succulentus* with centrally or laterally stipitate basidiomata, smaller stipe (up to 1 cm in length), smaller basidia (10.0–15.0 × 5.0–8.5 µm), and presence of gloeoplerous hyphae in tube tissues in contrast to the new species [14]. *Amylosporus campbellii* and *A*. *wadinaheezicus* share the terrestrial growth habit and basidiomata color. Both species were found under grasses, with more or less circular pileus [46]. *Amylosporus campbellii* is discernible from the new species by its (i) gloeoplerous hyphae present in both context and tube tissues, and (ii) substantially smaller basidia, measuring 10.0–20.0 × 7.0–8.5 µm. *Amylosporus sulcatus* with annual to perennial basidiomata associated with dead trunk of angiosperms, pileus surface snow white, pale pinkish cinnamon to pale brownish, pore surface snow white to pale brownish, gloeoplerous hyphae present in both contextual and stipe tissues, basidia 27.0–80.0 × 6.0–12.0 µm, 4-spored, clavate, with basal simple septum, basidiospores ellipsoid to ovoid, hyaline, measuring 3.6–5.7 × 2.7–3.6 µm [18]. *Amylosporus guaraniticus* has pileate sessile basidiomata, associated with underground log, with semicircular pileus, cap surface whitish to pale brownish or dark brown, pore surface creamy to light brown, gloeoplerous hyphae present in contextual tissues, basidia clavate, 4-spored, hyaline, smaller, measuring 10.0–25.0 × 5.0–8.0 µm, basidiospores ellipsoid, hyaline, finely asperulate, 4.0–5.5 × 3.0–4.0 µm [17]. *Amylosporus daedaliformis* with characteristic daedaliform shaped pores, gloeoplerous hyphae only present in tramal tissues, unbranched skeletal hyphae, basidiospores ellipsoid, smooth, measuring 5.0–6.0 × 2.5–3.5 µm [50].

#### 3.3.4. Key to the Known Species of *Amylosporus*

A taxonomic key to the known species of *Amylosporus* is presented below. This key is based on the growth habit of basidiomata (pileate or resupinate) and the presence or absence of clamp connections in generative hyphae. These are the two main morphological characters for the recognition of the proposed sections in the genus.

1. Growth habit of basidiomata pileate, and presence of clamps in generative hyphae—*Amylosporus* sect. *Amylosporus.* 2
  - Growth habit of basidiomata resupinate or effused-reflexed—*Amylosporus* sect. *Resupinati*. 7

2. Basidiomata annual or perennial, gloeoplerous hyphae present in both contextual and stipe tissues with large basidia measuring 27.0–80.0 × 6.0–12.0 µm. *A. sulcatus*
  - Basidiomata only annual. 3

3. Basidiomata eccentrically stipitate, pore surface buff clay, no. of pore 3–6 per mm, will smallest basidia in the genus measuring 10.0–15.0 × 6.0–8.0 µm. *A. auxiliadorae*
  - Basidiomata pileate stipitate or sessile, pore surface creamy-white to brownish. 4

4. Basidiomata laterally stipitate, circular to semicircular, appearing in lawn, stipe up to 1 cm in length, pores 2–4/mm, basidiomata watery. *A. succulentus*
  - Basidiomata sessile. 5

5. Basidiomata sessile, imbricate to semicircular, brownish pore surface, associated with buried logs. *A. guaraniticus*
  - Basidiomata stipitate. 6

6. Basidiomata sessile or stipitate, pores daedaliform shape at maturity, basidiospores smooth, measuring 5.0–6.0 × 2.5–3.5 µm. *A. daedaliformis*
  - Basidiomata centrally stipitate, gloeoplerous hyphae only present in context, absent in tube tissues, basidia larger, measuring 40–58 × 7–9 µm. *A. wadinaheezicus*

7. Generative hyphae septate and with multiple clamp connections. *A. bracei*
  - Generative simple septate and without clamp connection. 8

8. Basidiomata thin, less than 5 mm thick. *A. efibulatus*
  - Basidiomata thick, greater than 5 mm thick. 9

9. Basidiomata perennial, basidia eight-spored. *A. annosus*
  - Basidiomata annual, basidia four-spored. 10

10. Basidiospores up to 4 µm long, 3.5–4.0 × 2.7–3.2 µm. *A. casuarinicola*
  - Basidiospores > 4 µm long. 11

11. Basidiospores 4.0–6.0 × 3.0–4.2 µm, Q values 1.33 to 1.42. *A. rubellus*
  - Basidiospores 4.0–5.0 × 2.5–3.0 µm, Q value 1.6. *A. ryvardenii*

## 4. Discussion

Bondarzewiaceae is an ecologically diverse family in Russulales; some of the members are forest tree pathogens, while the majority of the species are wood-rotting fungi [2,3,4,5,6,7]. Currently, the family comprises nine genera and approximately 90 species, where most of these genera are associated with gymnosperm wood; however, genera like *Amylosporus* and *Stecchericium* are exclusively associated with angiosperm wood [2,3,4,5,6,13].

In the current taxonomy, Bondarzewiaceae consisted of *Amylaria*, *Amylosporus*, *Bondarzewia*, *Heterobasidion*, *Gloiodon*, *Laurilia*, *Lauriliella*, and *Wrightoporia* [8]. Among these genera, the systematic position of *Amylosporus* and *Wrightopori* has been in flux. Because *Wrightopori* was a polyphyletic genus, and both *Amylosporus* and *Wrightoporia* have been treated as unclassified genera in Russulales [2]. Some studies classified both the genera in Wrightoporiaceae [6,13,14,15], while some studies placed *Wrightoporia* in Wrightoporiaceae and *Amylosporus* as an unassigned genus in Russulales [54]. In the current study, the phylogenetic analyses of the family were evaluated based on combined ITS-28S sequence data of all genera of the family (except *Amylaria*, for which sequence data are unavailable). Two major clades were revealed using ML, BI, and time divergence estimation. One major clade consisting of *Amylosporus*, *Stecchericium*, and *Wrightoporia austrosinensis* with moderate support in ML analysis (BT 85%) and excellent support in BI analysis (PPs 0.96). Species in this major clade are associated with conifers, except *Wrightoporia austrosinensis*. The other major clade consisted of *Bondarzewia*, *Heterobasidion*, *Gloiodon*, *Laurilia*, *Lauriliella*, and *Wrightoporia*. The monotypic genus *Laurilia sulcata*, three species of *Gloidon* such as *G. nigrescens* (Petch) Maas Geest., *G. stratosus* (Berk.) Banker, *G. strigosus* (Sw.) P. Karst., and some species of *Bondarzewia* are associated with angiosperm wood [55,56]. However, the majority of the species in other genera of the family are exclusively associated with conifers [6,13,57].

### 4.1. Ecology and Distribution of Species of the Genera of Bondarzewiaceae

#### 4.1.1. *Amylaria* Corner

*Amylaria* is a monotypic, clavorioid genus with a single species, *A. himalayensis* Corner. The taxon is only known from Bhutan and Nepal [58]. The samples were found on the forest floor, around 2800 m in Nepal and 3000 m in Bhutan, among moss or on rotting buried wood at the base of a conifer tree [59]. The genus has been placed in *Bondarzewiaceae* according to the recent systematics reports [7]. Since no sequence data are unavailable and no report of the genus has been made after the original description, we think the systematic position of the taxon in Bondarzewiaceae is questionable [59].

#### 4.1.2. *Amylosporus* Ryvarden

There are 13 known species and one proposed new species in the genus *Amylosporus*. There are two main morphological characters: (i) the growth habit of basidiomata (pileate or resupinate) and (ii) the presence or absence of clamp connections in generative hyphae. Both these characters were employed for recognition of the proposed sections in the genus. The distribution and ecological association of each species in the genus are already discussed in the Taxonomy section. One species in section *Resupinati, Amylosporus bracei*, has clamp connections in generative hyphae, indicating that this character evolved twice during the course of the evolution of *Amylosporus*. The new species *Amylosporys wadinaheezicus* is most probably associated with underground wood, or it only grows on roots of *Anogeissus dhofarica*; perhaps it could be an endemic species to this region of the Arabian Peninsula.

#### 4.1.3. *Bondarzewia* Singer

In *Bondarzewia*, there are 16 species according to Index Fungorum (https://www.indexfungorum.org/names/Names.asp; accessed on 22 July 2024). The genus is characterized by pileate stipitate to substipitate basidiomata, and a dimitic hyphal system, the presence of lactiferous hyphae, with ornamented and amyloid basidiospores [60]. Some species in the genus are edible and have medicinal potential, such as *Bondarzewia mesenterica* (Schaeff.) Kreisel, according to the list of World’s Edible Mushroom [61]. Species in the genus are mostly associated with conifers; however, some are hosted by angiosperm trees. *Bondarzewia mesenterica* is the type species of genus. The species that are associated with flowering trees include *Bondarzewia berkeleyi* (Fr.) Bondartsev and Singer, distributed in temperate areas of eastern North America and Europe, associated with Fagaceae as tree pathogens [6], *B. dickinsii* (Berk.) Jia J. Chen, B.K. Cui, and Y.C. Dai has been reported from Japan to eastern China, associated with the fallen wood of *Quercus* species and the roots of *Castanea* species [62]; *B. guaitecasensis* (Henn.) J.E. Wright is a South American species associated with *Nothofagus* [63,64], *B. kirkii* J.A. Cooper, Jia J. Chen, and B.K. Cui reported from New Zealand, associated with the roots of *Fuscopora fusca* [62]. Other species, such as, *B. occidentalis* Jia J. Chen, B.K. Cui, and Y.C. Dai; *B. podocarpi* Y.C. Dai and B.K. Cui; *B. propria* (Lloyd) J.A. Cooper; *B. retipora* (Cooke) M.D. Barrett; *B. submesenterica* Jia J. Chen, B.K. Cui, and Y.C. Dai; *B. tibetica* B.K. Cui, J. Song, and Jia J. Chen; and *B. zonata* K. Das, A. Parihar and Hembrom are associated with gymnosperms [60,62,65,66]. Some species, such as *B. mesofila* R. Valenz., Baut.-Hern. and Raymundo are recently described species from tropical Mexico, which grow in the soil as saprotrophic mushrooms [67].

#### 4.1.4. *Gloiodon* P. Karst

*Gloiodon* is a small, hydnoid mushroom genus with resupinate or effused-reflexed basidiomata, with four known species. *Gloiodon occidentalis* Ginns has been reported from Canada, associated with the dead wood of *Tsuga heterophylla* [55]. The other species, such as *G. nigrescens* (Petch) Maas Geest., *G. stratosus* (Berk.) Banker, and *G. strigosus* (Sw.) P. Karst., are associated with angiosperm wood [55,56].

#### 4.1.5. *Heterobasidion* Bref.

*Heterobasidion* is an important white rot pathogenic poroid fungi, characterized by effused-reflexed to sessile basidiomata, dimitic hyphal system with dextrinoid skeletal hyphae, generative hyphae without clamp connections, and finely asperulate and nonamyloid basidiospores, distributed in the Northern and Southern Hemisphere [57]. The genus consisted of 18 species, mainly associated with conifers [57]. *Heterobasidion abietinum* Niemelä and Korhonen, distributed in Italy, associated with *Abies alba* and *Picea abies*; *H. amyloideum* Y.C. Dai, Jia J. Chen and Korhonen with *Abies* in Tibet China; *H. annosum* (Fr.) Bref. from Italy and Russia, associated with different species *Pinus*; *H. araucariae* P.K. Buchanan with trees of *Araucaria cunninghamii*, reported from Australia; *H. armandii* Y.C. Dai, Jia J. Chen and Yuan Yuan with *Pinus armandii*, found in China; *H. australe* Y.C. Dai and Korhonen from China, associated with *Pinus* species; *H. insulare* (Murrill) Ryvarden from China in association with *Pinus massoniana*; *H. irregulare* Garbel. and Otrosina is a South American species associated with *Pinus* species; *H. linzhiense* Y.C. Dai and Korhonen is a Chinese species associated with trees of *Abies* and *Picea* species; *H. occidentale* is pathogenic to various trees of conifers; *H. orientale* Tokuda, T. Hatt. and Y.C. Dai associated with fallen conifer trunk, reported from China; *H. parviporum* Niemelä and Korhonen associated with *Picea abies*, distributed in Europe and Asia; *H. subinsulare* Y.C. Dai, Jia J. Chen and Yuan Yuan is a recently reported species from China, associated with wood of *Pinus* species; *H*. *subparviporum* Y.C. Dai, Jia J. Chen and Yuan Yuan with wood of *Abies* and *Picea*, reported from China; and *H. tibeticum* Y.C. Dai, Jia J. Chen and Korhonen with *Pinus* wood from China [57].

#### 4.1.6. *Laurilia* Pouzar

*Laurilia* is a monotypic genus with *L. sulcata* (Burt) Pouzar, characterized by effuse-reflexed basidiomata with smooth to tuberculate hymenophore, trimitic hyphal system [54]. *Laurilia sulcata* is a widely distributed species in boreal conifer forests in the northern hemisphere [54].

#### 4.1.7. *Lauriliella* S.H. He and Nakasone

*Lauriliella* is a perennial genus with effused-reflexed, pileate or umbonate, woody basidiomata, hymenophore smooth to tuberculate, basidia with basal clamp connections, basidiospores broadly ellipsoid to subglobose, hyaline, thick-walled, echinulate, and amyloid [54]. The genus comprises two species, namely, *L. taxodii* (Lentz and H.H. McKay) S.H. He and Nakasone, and *L. taiwanensis* S.H. He and Nakasone. *Lauriliella taxodii* are distributed in USA, causing white stringy rot or brown powdery rot in living *Taxodium distichum*. Similarly, *L. taiwanensis* is reported from Taiwan, causing white rot in living *Chamaecyparis formosensis* [54].

#### 4.1.8. *Stecchericium* D.A. Reid

*Stecchericium* with dimitic hyphal system, clamped generative hyphae and skeletal hyphae, variable types of cystidia in the hymenium, and amyloid basidiospores [7]. Species of the genus are wood decaying mushrooms, associated with angiosperms, currently with six species, and typified with *Stecchericium seriatum* (Lloyd) Maas Geest. [7]. According to MycoBank, this genus belongs to Wrightoporiaceae; however, recent studies [7,8,9] and Index Fungorum classified *Stecchericium* in Bondarzewiaceae. The known species in the genus are: *Stecchericium abditum* Maas Geest., found on rotten log in Australia [68], *S. acanthophysium* T. Hatt. and Ryvarden on hardwood reported from Japan [69], *S. isabellinum* Corner associated with fallen wood in the Amazon forest [70], and *S. rusticum* Maas Geest., on dead wood in Singapore [71]. *Stecchericium fistulatum* (G. Cunn.) D.A. Reid, which was the type species of the genus, is now considered a synonym of *S. seriatum* (Lloyd) Maas Geest. [72]. *Stecchericium dimiticum* Douanla-Meli is associated with angiosperm wood, reported from Cameroon [73].

#### 4.1.9. *Wrightoporia* Pouzar

*Wrightoporia* is a large and diverse genus of wood decaying tropical polypores and has been shown to be polyphyletic [6]. The systematic position of the genus has been in flux; initially it was placed in *Wrightoporiaceae* [74], transferred to *Hericiaceae* [53], and according to Index Fungorum and recent studies, the genus has been placed in *Bondarzewiaceae* [7,8,9]. However, MycoBank still considered *Wrightoporia* in Wrightoporiaceae [accessed on 20 July 2024]. The type species of the genus is *Wrightoporia lenta* (Overh. and J. Lowe) Pouzar [75]. The generic circumscription of the genus has recently been revised [13]. According to the recent definition, *Wrightoporia* s.str. is distinguished by an annual growth pattern, resupinate to effused-refluxed basidiomata, soft and cottony at fresh condition, membranous to cottony at dry condition, pores rounded to angular, 1–4/mm, margins mostly having rhizomorphs, skeletal hyphae narrow with 0.8–2.5 µm in diam, basidiospores finely asperulate, mostly found on dead logs of gymnosperms, rarely on angiosperms. Currently, there are six species in the genus, distributed in Asia: *Wrighoporia austrosinensis* Y.C. Dai, *W. avellanea* (Bres.) Pouzar, *W. borealis* Y.C. Dai, *W. lenta*, *W. srilankensis* Y.C. Dai and Yuan Yuan, and *W. subavellanea* Jia J. Chen and B.K. Cui [13,76,77]. Cysidia is absent in these species; however, in *Wrightoporia austrosinensis*, cystidia and cystidiols are present [78]. Interestingly, in a recent phylogenetic analysis, *W. austrosinensis* formed an independent lineage outside of the genus [77]. A similar phylogenetic position of *W. austrosinensis* outside of *Wrightoporia* was observed during this study. This species could not be a member of *Wrightoporia*; perhaps it could be included either in *Amylosporus* or may be treated as an independent genus.

## 5. Conclusions

It is concluded from this study that members of *Bondarzewiaceae* are associated with angiosperm or gymnosperm wood, causing white rot in tree species. Microscopically, the species with dimitic hyphal system except *Laurilia* with trimitic hyphal system. *Wrightoporia austrosinensis* is not a species in *Wrightoporia*; it is suggested that it could be an independent genus or a species in *Amylosporus*.

## Figures and Tables

**Table 1:** Taxa used in phylogenetic analyses, presented here as appeared in Figure 2 and Figure 3, bold fonts represent the new species.

Genus/Section	Species	Herbarium Voucher	Country of Origin	GenBank Accession	
				ITS	28S
*Amylosporus* sect. *Amylosporus*	***Amylosporus wadinaheezicus***	**JHN-22-006**	**Oman**	**PP681310**	**PP681316**
	***Amylosporus wadinaheezicus***	**SALALAH-002**	**Oman**	**PP697985**	**PP681315**
	***Amylosporus wadinaheezicus***	**JHN-22-009**	**Oman**	**PP681311**	**PP681314**
	***Amylosporus wadinaheezicus***	**NHZ-22-004**	**Oman**	**PP681308**	**PP681313**
	*Amylosporus sulcatus*	GXU 1084	China	MG280818	MG280819
	*Amylosporus sulcatus*	GXU 1095	China	MG280820	MG280821
	*Amylosporus* sp.	IJ-2014_IJV29-2	Tanzania	KM851314	KM593892
	*Amylosporus* sp.	IJ-2014_IJV29-1	Tanzania	KM851315	KM593893
	*Amylosporus campbellii*	0806-20a	Jamaica	JF692200	KJ807077
	*Amylosporus campbellii*	Gilbertson_14806	USA	KM107861	KM107879
	*Amylosporus guaraniticus*	M._Campi_106	Paraguay	MF377528	MF377529
	*Amylosporus guaraniticus*	M._Campi_105	Paraguay	MF377530	-
	*Amylosporus succulentus*	Dai_7802	China	KM213669	KM213671
	*Amylosporus succulentus*	Dai_7803	China	KM213668	KM213670
*Amylosporus* sect. *Resupinati*	*Amylosporus annosus*	BJFC-Dai_18640	Malaysia	MH647059	MH647055
	*Amylosporus annosus*	BJFC-Dai_18638	Malaysia	MH647058	MH647054
	*Amylosporus annosus*	BJFC-Dai_18635	Malaysia	MH647057	MH647053
	*Amylosporus annosus*	BJFC-Dai_18632	Malaysia	MH647056	MH647052
	*Amylosporus* sp.	JV_1809-4	USA	MN888695	MN888696
	*Amylosporus rubellus*	Dai_9233	China	KJ807071	KJ807084
	*Amylosporus casuarinicola*	Dai_6914	China	KJ807068	-
	*Amylosporus casuarinicola*	Yuan_1614	China	KM107862	-
	*Amylosporus bracei*	1008-77	USA	KM267724	KJ807076
	*Amylosporus* sp.	Dai_22165	China	OL473603	OL473616
*Wrightoporia s.l.*	*Wrightoporia austrosinensis*	UC2023009	USA	KP814178	-
	*Wrightoporia austrosinensis*	Dai 11579	China	KJ807065	KJ807073
*Stecchericium*	*Stecchericium seriatum*	CBS:756.81	Singapore	MH861476	-
	*Stecchericium seriatum*	SL1685	Singapore	OR527392	-
*Bondarzewia*	*Bondarzewia podocarpi*	Dai 9261	China	KJ583207	KJ583221
	*Bondarzewia propria*	PDD 60293	New Zealand	KJ583213	KJ583227
	*Bondarzewia occidentalis*	HHB 14803	USA	KM243329	KM243332
*Lauriliella*	*Lauriliella taiwanensis*	FP-101635	China: Taiwan	KY172891	KY172906
	*Lauriliella taxodii*	FP-105464-Sp	USA	KY172896	KY172912
*Laurilia*	*Laurilia sulcata*	He 20120916-7	China	KY172894	KY172909
	*Laurilia sulcata*	KHL 8267	Russia	AF506414	AF506414
*Heterobasidion*	*Heterobasidion amyloideum*	Cui 12656	China	MT146480	MT446029
	*H. armandii*	Dai 17605	China	MT146482	MT446031
	*Heterobasidion abietinum*	00053/1	Italy	KJ651451	KJ651509
*Gloiodon*	*Gloiodon nigrescens*	Desjardin7287	Bali	AF506450	AF506450
	*Gloiodon strigosus*	JS26147	Norway	AF506449	AF506449
*Wrightoporia s.str.*	*Wrightoporia srilankensis*	Dai_19621	Sri Lanka	MN688691	MN688684
	*Wrightoporia srilankensis*	Dai_19667	Sri Lanka	MN688692	MN688685
	*Wrightoporia srilankensis*	Dai_19500	Sri Lanka	MN688690	MN688683
	*Wrightoporia srilankensis*	Dai_19668	Sri Lanka	MN688693	MN688686
	*Wrightoporia srilankensis*	Dai_19620	Sri Lanka	MN688694	MN688687
	*Wrightoporia avellanea*	LR41710	Jamaica	AF506488	AF506488
	*Wrightoporia lenta*	Dai 10462	China	KJ513291	KJ807082
	*Wrightoporia lenta*	Cui_7804	China	KJ513292	KJ807081
	*Wrightoporia subavellanea*	Dai_11484	China	KJ513295	KJ807085
	*Wrightoporia subavellanea*	Dai_11488	China	KJ513296	KJ807086
	*Wrightoporia subavellanea*	Dai_11492	China	KJ513297	KJ807087
Outgroup	*Amylonotus labyrinthinus*	Yuan 1475	China	KM107860	KM107878

## Data Availability

The original contributions presented in the study are included in the article, further inquiries can be directed to the corresponding authors.
